# The power of the collective empowers women: Evidence from self-help groups in India

**DOI:** 10.1016/j.worlddev.2021.105579

**Published:** 2021-10

**Authors:** Neha Kumar, Kalyani Raghunathan, Alejandra Arrieta, Amir Jilani, Shinjini Pandey

**Affiliations:** a International Food Policy Research Institute, Washington, DC, United States; b International Food Policy Research Institute, New Delhi, India; c University of Washington, Department of Health Metrics Sciences, United States; d Asian Development Bank, Manila, Philippines; e Department of Agricultural, Environmental, and Development Economics, Ohio State University, Columbus, Ohio, United States

**Keywords:** Women, Self-help groups, India, Empowerment, Gender

## Abstract

Women’s groups are important rural social and financial institutions in South Asia. In India, a large majority of women’s groups programs are implemented through self-help groups (SHGs). Originally designed as savings and credit groups, the role of SHGs has expanded to include creating health and nutrition awareness, improving governance, and addressing social issues related to gender- and caste-based discrimination. This paper uses panel data from 1470 rural Indian women from five states to study the impact of SHG membership on women’s empowerment in agriculture, using the project-level Women’s Empowerment in Agriculture Index (pro-WEAI) and the abbreviated Women’s Empowerment in Agriculture Index (A-WEAI). Because SHG membership was not randomized and women who self-select to be SHG members may be systematically different from non-members, we employ nearest neighbor matching methods to attribute the impact of SHG membership on women’s empowerment in agriculture and intrahousehold inequality.

Our findings suggest that SHG membership has a significant positive impact on aggregate measures of women’s empowerment and reduces the gap between men’s and women’s empowerment scores. This improvement in aggregate empowerment is driven by improvements in women’s scores, not a deterioration in men’s. Greater control over income, greater decisionmaking over credit, and (somewhat mechanistically, given the treatment) greater and more active involvement in groups within the community lead to improvements in women’s scores. However, impacts on other areas of empowerment are limited. The insignificant impacts on attitudes towards domestic violence and respect within the household suggest that women’s groups alone may be insufficient to change deep-seated gender norms that disempower women. Our results have implications for the design and scale-up of women’s group-based programs in South Asia, including the possibility that involving men is needed to change gender norms.

## Highlights

• We study the impacts of women’s Self-help group membership on women’s and men’s empowerment.
• We measure empowerment using two alternate indices: the A-WEAI and the Pro-WEAI.
• We find that SHG membership has a significant positive impact on aggregate measures of women’s empowerment.
• We also find that SHG membership reduces the gap between men’s and women’s empowerment scores.
• The impacts are driven by increase in control over income, decisionmaking over credit, and active involvement in groups.

## 1 Introduction

Over the past three decades, women’s groups have rapidly gained prominence as rural social and financial institutions, particularly in South Asia. In India, many women’s groups programs are implemented through self-help groups (SHGs). SHGs are groups of 10–20 women that meet at regular intervals to deposit money into a group-held account from which loans can be requested in times of need. These groups are typically formed by women who live close to one another and are ethnically and economically homogenous (Baland et al., 2011, Sharma, 2001). SHGs reaching maturity are provided linkages to bank accounts and lines of credit, federated into higher-order collectives, and provided a range of other inputs – information on agricultural practices, inputs into these practices, trainings on livelihoods activities and so on - depending on the government entity or nongovernmental organization (NGO) that facilitates their formation. More recently, their role has expanded to include creating health and nutrition awareness, generating demand for various government programs, ensuring transparency in the implementation of government schemes, and tackling social issues ranging from dowry and domestic violence to gender- and caste-based discrimination (Chen, Jhabvala, Kanbur, & Richards, 2007 cited in Desai and Joshi, 2013, Kumar et al., 2019).

This paper uses data on poor rural men and women from five states in eastern and central India to study the impacts of group membership on women’s empowerment. We use two variations of the Women’s Empowerment in Agriculture Index (the WEAI) to estimate the effect of SHG membership on the level of women’s empowerment, and her empowerment relative to that of her partner. We find that SHG membership positively and significantly affects women’s overall empowerment score and reduces the gap in empowerment scores between the couple. While SHG membership has strong impacts on control over income and decisionmaking, among other outcomes, it does not affect other forms of empowerment that are driven by gender norms that might be harder to shift, such as attitudes towards domestic violence and respect within the household. Reassuringly, reductions in the empowerment score gap are not driven by the disempowerment of men.

SHG programs in India have deliberately targeted women not only due to their low status in society relative to men, but also because women’s SHGs have proved to be successful and sustainable (Parida & Sinha, 2010). As a result of their reach – these groups currently reach more than 50 million households across the country (NRLM, 2020) – donors and policymakers are increasingly interested in using SHGs as platforms for service delivery, as collateral substitutes that help to build other forms of capital, and as vehicles for women’s empowerment. Kumar et al. (2018) develop a conceptual framework linking women’s group-based programs to health and nutrition outcomes among women and assert that women's groups can be socially empowering; they help build social capital by creating a comfortable space where women can voice their opinions and share experiences with fellow members, often achieved through group-building exercises that teach listening skills, build trust, and enhance participation. Many group-based programs also lend themselves to interventions that emphasize collective action. For example, using group platforms to deliver participatory learning and action (PLA) strategies enables members to effectively identify shared problems, plan strategies, act together, and assess impacts (Harris-Fry et al., 2016, Kanani et al., 2015); this approach is distinguished from others that simply transmit information to members but do not necessarily build community capacity to act collectively.

The evidence on whether these groups do indeed improve women’s empowerment is, however, somewhat mixed. A recent systematic review by Brody et al. (2017) found that women’s economic SHGs have positive effects on economic and political empowerment, women’s mobility, and women’s control over family planning, although the authors did not find evidence of positive effects of SHGs on psychological empowerment. Qualitative evidence from rural Nepal found that increased access to funds through community women’s groups did increase women’s independence and decisionmaking to some extent (Morrison et al., 2010); Morrison et al., 2019 also find that PLA approaches with women’s groups in four countries – Bangladesh India, Nepal and Malawi – increased women’s confidence to negotiate with family members around adoption of recommended behaviors. In contrast, a review of the (largely South Asia focused) literature on the impact of group-based community mobilization efforts on women and children’s health included several qualitative and quantitative studies that reported increases in self-confidence or self-efficacy but concluded that the overall quality of evidence on this aspect was low (Gram, Fitchett, Ashraf, Daruwalla, & Osrin, 2019a). In a multi-arm study of pregnant women’s groups in rural Nepal, Gram, Morrison et al (2019b) find limited evidence of improvements in women’s agency as a result either of a participatory learning and action (PLA) approach alone or of PLA combined with food or cash transfers. In the context of participatory women’s groups in rural Nepal, Gram, Morrison et al., 2019b looked at long-term impacts on women’s agency – measured using the Relative Autonomy Index – and found no impact either of initial or subsequent exposure to the PLA groups.

In fact, Gram, Morrison et al. (2019b) caution that women’s agency might be a prerequisite to the success of these groups, rather than a consequence of women’s participation. For women to benefit from any programmatic inputs, they need to have some level of control or agency over their own decisions and be respected within their communities. Strategies to empower women may include increasing their financial independence, encouraging them to run for elected positions in political offices or village councils, promoting a more gender-equitable division of household labor, building perceptions of autonomy and self-wellbeing, improving women’s negotiating skills with husbands, and increasing control over reproductive choices, among others. As the disempowered woman builds confidence and gains support from men and the community at large, she will be better able to make decisions that promote her own health and that of her children (Sraboni, Malapit, Quisumbing, & Ahmed, 2014).

Whether SHGs empower women in agriculture is important in the Indian context for several reasons. The first is one of scale. SHGs formed under the national government initiative – the National Rural Livelihoods Mission (NRLM) – already cover approximately 48 million households, and plan to reach 100 million households by 2024 (Ministry of Rural Development, 2018). Significant resources have been and continue to be invested both by government and NGOs to form and strengthen SHGs.

Second, women in India fare worse than men along several indicators. The female adult literacy rate is just 59% (versus that for males at 79%), women form only 24.6% of the total labor force, and women farmers control <13% of total operational holdings (World Bank, 2011, Agriculture Census, 2010-11). Given their low status in society, empowering women is, of course, intrinsically valuable. In addition, some studies have documented linkages with other development goals, such as eliminating poverty, reducing hunger and malnutrition, and achieving good health and well-being for women and their families (Cunningham et al., 2015, Malapit et al., 201a, Malapit et al., 2015, Ruel et al., 2018, Sraboni et al., 2014), though the broader evidence base for low and middle-income countries is mixed (Harris-Fry et al., 2020), and certain time-intensive interventions could have unintended consequences by competing with caregiving, rest or domestic chores like food preparation in certain contexts (Carlson et al., 2015, Johnston et al., 2015, Komatsu et al., 2018).

Third, agriculture is the largest sector in India, employing almost half the population (Gillespie et al., 2012, Planning Commission, 2007, Census, 2011), but women farmers control a very small proportion of total landholdings and are often unreached by government systems of information and agricultural extension. As a result, women farmers are often unable to access the information needed to make decisions around production, or to enhance their productivity by adopting newer and more innovative methods. Women farmers from historically marginalized social groups – Scheduled Castes (SCs) and Scheduled Tribes (STs) – are doubly constrained, as the female and caste disadvantages reinforce each other: a study on gender and agriculture extension in India (World Bank and IFPRI, 2010) found that villages represented by female *gram panchayat* members from scheduled castes received significantly fewer resources, such as agricultural extension services, than others. These gender-based inequities are, of course, not limited to India, with women in several contexts having lower asset ownership, lower labor force participation and greater work-related burdens (Harris-Fry et al., 2020, Ruel et al., 2018, Doss et al., 2015, Palacios-López and López, 2015). These inequities often serve to limit the ability of agricultural interventions to bolster household incomes and food security and ensure equitable access to nutritious diets (Harris-Fry et al., 2020). For all these reasons, studying the impact of SHGs on women’s empowerment in agriculture - particularly those SHGs that target marginalized caste and tribe groups - could have wide-ranging policy implications in India.

We use data from an impact evaluation of a nutrition-sensitive agriculture intervention being implemented in five states in India by Professional Assistance for Development Action (PRADAN), one of India’s largest NGOs, to study the impact of SHG membership on women’s empowerment in agriculture. PRADAN works to form, strengthen, and support women’s SHGs, often independently but on occasion in collaboration with the National or State-specific Rural Livelihoods Missions. The broader nutrition-sensitive agriculture intervention being evaluated was delivered through the PRADAN SHG platform, but other organizations, both NGO- and government-led, work in these study areas to form and support SHGs, resulting in a mix of PRADAN and non-PRADAN groups. We use a subsample drawn from within the sample of individuals selected for inclusion in the broader evaluation and look specifically at membership in *any* SHG – either PRADAN- or government- or NGO-supported – on women’s empowerment measures. Thus, while nested within the context of a larger impact evaluation, the data for this study should be viewed as a stand-alone dataset. For this study, we use two recently developed measures of women’s empowerment: the project-level Women’s Empowerment in Agriculture Index (pro-WEAI), which was designed in collaboration with 13 agricultural development projects for project use (Malapit et al., 2019, Malapit et al., 2019), and an abbreviated version of a recent internationally validated measure of empowerment, the A-WEAI (Malapit, Pinkstaff, Sproule, Kovarik, Quisumbing, & Meinzen-Dick, 2017), which was designed for large-scale, population-based surveys. Both indexes are modifications of the Women’s Empowerment in Agriculture Index (WEAI) (Alkire et al., 2013), and, like the WEAI, are based on interviews of the primary male and female adults in the same household. Both pro-WEAI and the A-WEAI provide an aggregate measure of empowerment as well as a measure of intrahousehold empowerment gaps. Because they are additive, decomposable Alkire and Foster (2011) indices, they can be decomposed into their component indicators to understand specific pathways of impact. Comparing the results from both the indices also allows us to analyze what can be learned from additional indicators that were chosen by projects themselves for inclusion in the pro-WEAI.

Our analysis is based on panel data collected in 2015 and 2017 from 1470 households across five states in India. These 1470 households are drawn from the larger impact evaluation study sample and include those for which we have information both on membership in any SHG (PRADAN or non-PRADAN) and on women’s empowerment. Because access to SHG membership in our sample was not random and women who self-select to join SHGs may be systematically different from those who do not, we employ nearest neighbor matching (NNM) methods to attribute causality. We analyze the impact of SHG membership on overall empowerment measures at the individual and household level - the aggregate empowerment score and the empowerment gap - and then unpack these results by looking at the component indicators for each empowerment domain. Wherever relevant, impacts are estimated for both women and men. In the case of men, comparisons are made between men whose partners are SHG members and those whose partners do not belong to an SHG.

We find that SHG membership has a significant positive impact on aggregate measures of women’s empowerment and reduces the gap in empowerment scores of women and men within the same household. Results are qualitatively similar whether we use pro-WEAI or A-WEAI. Higher levels of aggregate empowerment for women SHG members is driven by greater control over income, greater decisionmaking over credit, and greater and more active involvement in groups within the community. However, impacts on production decisions and asset ownership are limited, and the weakly significant and negative impacts on workloads indicate that group membership may involve tradeoffs in terms of time use. While SHG members can go to more places than nonmembers—owing possibly to the need to attend group activities—their mobility remains limited. The insignificant impacts on other measures of empowerment related to attitudes towards intimate partner violence and respect within the household suggests that, despite impacts on some measures of empowerment, being an SHG member may not be enough to change deep-seated gender norms that disempower women. Finally, and perhaps reassuringly, greater empowerment of women in some areas and the smaller empowerment gap between spouses does not appear to come from men’s disempowerment. Except for the credit domain, where men married to SHG members also have greater participation in credit-related decisions, the impact of having a wife who belongs to an SHG on men’s empowerment outcomes is small and largely insignificant.

Our findings add to the small but growing literature on the impact of women’s groups on various aspects of empowerment. The literature on social capital has long pointed to the importance of women’s groups as pathways towards empowerment (for a review, see Meinzen-Dick, Behrman, Pandolfelli,Peterman, & Quisumbing, 2014), but the specific pathways of impact have been studied only relatively recently, following the growth and expansion of these platforms for service delivery in South Asia. Brody et al. (2017)’s systematic review of the literature on SHGs in South Asia and their impact on women’s empowerment identifies three immediate outcomes of group membership - improved access to credit, training and other resources provided through the groups, exposure to group support, and the accumulation of social capital, which in turn lead to intermediate outcomes of increased income and savings, reduced debt, and increased autonomy and self-efficacy. These, in turn, can result in both positive and adverse long-term impacts – the increased ability to translate choices into actions but also the possibility of increased tension within the household, stigma and backlash from the community or a greater incidence of domestic violence. Reassuringly, their review of the evidence from qualitative and quantitative studies suggests that while SHGs improve women’s economic and political empowerment, their mobility, and their control over family planning, there is no evidence of an adverse effect on the incidence of domestic violence.

Using retrospective data from the JEEViKA SHG-based project in Bihar, Datta (2015) finds evidence of reduced debt and increased mobility, decision-making and collective action for beneficiary households. These findings serve to corroborate those of Brody et al. (2017), although the data, being retrospective, is subject to recall bias.

In related work using baseline data from this study, Kumar et al. (2019) apply NNM methods to show that SHG members are more politically engaged than nonmembers, more aware of their rights and entitlements, and, perhaps owing to this increased awareness, more likely to have availed of a greater number of government entitlement schemes. SHG members also have wider social networks and display greater mobility than nonmembers. Using the same data, Raghunathan, Kannan, and Quisumbing (2019) find that SHG membership increases women’s access to information and their participation in some agricultural decisions, but has limited impact on agricultural practices or outcomes, possibly due to financial constraints, social norms, and women’s domestic responsibilities. To the best of our knowledge, the impact of SHG membership on the WEAI and its variants has not yet been studied in the context of South Asia, though several evaluations are currently underway.

The rest of this paper is organized as follows: Section 2 describes the measurement of women’s empowerment and how the WEAI and its adaptations can be used to assess the impacts of interventions. Section 3 describes the data used for analysis, and the empirical strategy. Section 4 presents the results from each of our specifications, and Section 5 concludes with a discussion of our findings.

## 2 Measuring empowerment

Measuring women’s empowerment poses a challenge, as it is a complex multidimensional concept that can be measured in many different ways (Agarwala and Lynch, 2006, Kabeer, 1999, Miedema et al., 2018, Pratley, 2016, Santoso et al., 2019). Much of the literature studying women’s empowerment has used proxy measures, like mobility, decision-making power over allocation of household resources, participation in political processes, strength of social networks, and so on. As Santoso et al. (2019) point out, differences in the ways in which these measures are collected and aggregated for use adds an additional layer of complexity to the study of women’s empowerment. In addition, these measures are not necessarily grounded in a theoretically robust definition of empowerment, and often rely on what is available in existing data sets, such as the Demographic and Health Surveys, which tend to focus on decisionmaking in the domestic and reproductive spheres. Ewerling et al. (2017) use DHS data from 34 countries to propose the Survey-based Women’s EmPowERment index or SWPER; while this provides a useful starting point, the construction of this index has been critiqued, and the suggestion of more thorough psychometric assessments is made (Yount, Peterman, & Cheong, 2018).

In fact, attempts to develop and formalize women’s empowerment measures that are based in theories of empowerment and the resources-agency-achievements framework laid out in Kabeer (1999) are fairly recent. This paper improves upon the existing literature by using two modifications of one such index – the Women’s Empowerment in Agriculture Index (WEAI) (Alkire et al., 2013), a survey-based, internationally validated measure of women’s empowerment in the agricultural sector. These modifications, discussed below, are the project-level WEAI (pro-WEAI) (Malapit et al., 2019, Malapit et al., 2019) and the abbreviated WEAI (A-WEAI) (Malapit et al., 201a, Malapit et al., 2015). All WEAI-based measures draw from Kabeer’s (1999) definition of empowerment as expanding people’s ability to make strategic life choices, particularly in contexts in which this ability had previously been denied to them. In this definition, the ability to exercise choice encompasses three dimensions: resources (not only access but also income and future claims to material, human, and social resources), agency (processes of decision-making, negotiation, etc.), and achievements (well-being outcomes, educational levels). The WEAI family of measures focuses on “agency” – far less studied than resources or achievements – because it directly addresses the issue of choice or decision-making.

Pro-WEAI and A-WEAI are similar indexes with some differences in emphasis. Pro-WEAI was developed by the Gender, Agriculture, and Assets Project, Phase 2 (GAAP2) in response to demand from implementors of agricultural development projects for an empowerment measure that captured aspects of empowerment relevant to the success of their projects and that was more closely linked to theories of agency.^1^ It covers three domains of intrinsic, instrumental and collective agency, and comprises 12 indicators that implementors thought were important aspects of project success (Malapit et al., 2019, Malapit et al., 2019). The A-WEAI, which was designed for implementation in population-based surveys, was developed in response to partners’ requests to reduce interview time and eliminate modules that were time-consuming, sensitive, and difficult to understand (Malapit et al., 201a, Malapit et al., 2015). In contrast to the pro-WEAI, A-WEAI covers five-domains and consists of six indicators. A-WEAI can be derived from pro-WEAI with a slightly different weighting structure and modified indicator cut-offs, and hence can be thought of as being nested in pro-WEAI. A comparison between the two indexes is presented in Table 1, with pro-WEAI indicators in the left column and A-WEAI indicators on the right.

**Table 1:** Domains and indicators of the project-level Women’s Empowerment in Agriculture Index (pro-WEAI) and the Abbreviated Women’s Empowerment in Agriculture Index (A-WEAI).

***Pro-WEAI Domain* Indicator**	**Weight in pro-WEAI**	**Adequacy definition**	**A-WEAI Domain Indicator**	**Weight in A-WEAI**
**Intrinsic agency**			n/a	n/a
Autonomy in income	1/12	More motivated by own values than by coercion or fear of others’ disapproval: *Relative Autonomy Index*^B^ score>=1 RAI score is calculated by summing responses to the three vignettes about a person’s motivation for how they use income generated from agricultural and non-agricultural activities (yes = 1; no = 0), using the following weighting scheme: 0 for vignette 1 (no alternative), −2 for vignette 2 (external motivation), −1 for vignette 3 (introjected motivation), and + 3 for vignette 4 (autonomous motivation)	n/a	n/a
Self-efficacy	1/12	“Agree” or greater on average with self-efficacy questions: *New General Self-Efficacy Scale*^C^ score>=32	n/a	n/a
Attitudes about intimate partner violence against women	1/12	Believes husband is NOT justified in hitting or beating his wife in all 5 scenarios:^d^ 1) She goes out without telling him 2) She neglects the children 3) She argues with him 4) She refuses to have sex with him 5) She burns the food	n/a	n/a
Respect among household members	1/12	Meets ALL of the following conditions related to their spouse, the other respondent, or another household member: 1) Respondent respects relation (MOST of the time) AND 2) Relation respects respondent (MOST of the time) AND 3) Respondent trusts relation (MOST of the time) AND 4) Respondent is comfortable disagreeing with relation (MOST of the time)	n/a	n/a
**Instrumental agency**			**Production**	
Input in productive decisions	1/12	Pro-WEAI definition: Meets at least ONE of the following conditions for ALL of the agricultural activities they participate in 1) Makes related decision solely, 2) Makes the decision jointly and has at least some input into the decisions 3) Feels could make decision if wanted to (to at least a MEDIUM extent) A-WEAI definition^a^: Adequate if individual participates in and makes decisions, has input in decisions, or feels she could make decisions (if desired) about at least one agricultural activity	Input in productive decisions	1/5
Ownership of land and other assets	1/12	Pro-WEAI definition: Owns, either solely or jointly, at least ONE of the following: 1) Any three assets 2) Land A-WEAI definition^a^: Adequate if individual owns at least one major asset or at least two minor assets	Ownership of land and other assets	2/15
Access to and decisions on financial services	1/12	Pro-WEAI definition: Meets at least ONE of the following conditions: 1) Belongs to a household that used a source of credit in the past year AND participated in at least ONE sole or joint decision about it 2) Belongs to a household that did not use credit in the past year but could have if wanted to from at least ONE source 3) Has access, solely or jointly, to a financial account A-WEAI definition^b^: Adequate if individual makes decisions about at least one source of credit accessed by her/his household	Access to and decisions on credit	1/15
			**Income**	
Control over use of income	1/12	Pro-WEAI definition: Has input in decisions related to how to use BOTH income and output from ALL of the agricultural activities they participate in AND has input in decisions related to income from ALL non-agricultural activities they participate in, unless no decision was made A-WEAI definition^a^: Adequate if individual participates in and has input in decisions about income generated from an activity or she/he makes decisions, has input in decisions, or feels she/he could make decisions (if desired) about employment or major household expenditures	Control over use of income	1/5
			**Time**	
Work balance	1/12	Pro-WEAI definition: Works <10.5 h per day: Workload = time spent in primary activity + (1/2) time spent in childcare as a secondary activity A-WEAI definition^c^: Adequate if individual worked<10.5 h during the previous day Workload = time spent in primary activity	Workload	1/5
Visiting important locations	1/12	Meets at least ONE of the following conditions: 1) Visits at least TWO locations at least ONCE PER WEEK of [city, market, family/relative], or 2) Visits least ONE location at least ONCE PER MONTH of [health facility, public meeting]	n/a	n/a
**Collective agency**			**Leadership**	
Group membership	1/12	Active member of at least ONE group	Group membership	1/5
Membership in influential groups	1/12	Active member of at least ONE group that can influence the community to at least a MEDIUM extent	n/a	n/a

*Notes:* n/a means “not applicable” and applies to pro-WEAI indicators that are not included in A-WEAI. Cells shaded in green are indicators that are common to both indices.

a pro-WEAI uses a stricter adequacy cut-off.

b pro-WEAI includes access to financial accounts.

c pro-WEAI includes only childcare as a secondary activity in the 24-hour recall module; secondary activities are not collected in A-WEAI.

d Calculation of overall measures: In A-WEAI, an individual is defined as empowered if she/he is adequate in 80% of the indicators as defined and weighted in Table 1. In pro-WEAI, we estimate two empowerment scores: (1) the individual is adequate in 70% of the indicators and (2) the individual is empowered in 80% of the indicators, in both cases as defined and weighted in Table 1. The overall 5DE (A-WEAI)/3DE (pro-WEAI) score is calculated as a composite index that measures two aspects of empowerment, a) the proportion of women who are empowered, and b) the average empowerment score among disempowered women. The second component of the A-WEAI and pro-WEAI indices - the GPI - is calculated in the same way for both, except that the two indices use different indicators and adequacy cut-offs to define empowerment, as laid out in Table 1.

In our study, we measure empowerment using the pro-WEAI and the A-WEAI. Both are aggregate indices composed of two sub-indices, the aggregate empowerment score and the gender parity index (GPI)^2^. In pro-WEAI, the empowerment score is defined over three domains and is hence called the 3DE (3 Domains of Empowerment); in A-WEAI, the empowerment score is defined over five domains and is called the 5DE. The 3DE, the first sub-index of the pro-WEAI, assesses whether individuals are empowered in the three domains of intrinsic, instrumental (“power to”) and collective (“power with”) agency. Intrinsic agency, or “power within”, assesses an individual’s sense of worth, self-confidence, and self-respect. This includes indicators for autonomy in income, self-efficacy, attitudes about intimate partner violence (IPV) against women, and respect among household members. Instrumental agency, or “power to”, measures an individual’s ability to make decisions in their own best interest and includes indicators for input in productive decisions, ownership of land and other assets, access to and decisions on financial services, control over use of income, work balance, and visiting important locations. Finally, collective agency, or “power with”, determines the power of association and includes indicators for group membership and membership in influential groups. The respondent is identified as being empowered in each of the 12 indicators based on pre-determined thresholds, or cutoffs. A simple equal weighting structure (Table 1) is used to aggregate scores from the 12 indicators in these three domains into the 3DE score. In the A-WEAI, a similar procedure is followed, but with different weights. Definitions of the three domains under pro-WEAI, the five domains under the A-WEAI, the corresponding indicators, the definition of adequacy, and their weights are presented in Table 1. The 3DE and 5DE scores, measured at the individual level, measure the extent to which an individual is empowered, with higher 3DE and 5DE scores indicating greater empowerment.

Because the same information is also collected from the respondent woman’s husband or partner (or a male decision maker if the partner is not available), 3DE and 5DE scores are also computed for the primary male. A comparison of these scores for the primary male and female within the same household is used to calculate the gender gap in empowerment, referred to as the intrahousehold inequality score. This gap is zero in households where the woman is empowered (irrespective of relative empowerment of the primary male and female respondent).^3^ For households where the woman is not empowered, the gender gap provides a measure of the gap in empowerment scores that needs to be closed for the woman to be as empowered as the man. The greater the gender gap, the greater the shortfall in women’s empowerment.

This intrahousehold empowerment gap is used to determine whether the household achieves gender parity in empowerment. This is aggregated into the second sub-index, the GPI, which measures the *relative* equality in empowerment of men and women at the sample level. The 3DE and 5DE contribute 90 percent of the weight to the pro-WEAI and A-WEAI, respectively, and the GPI contributes the remaining 10 percent. Further details on the computation of the two indexes are found in Malapit et al., 2019, Malapit et al., 2019, Malapit et al., 201a.

## 3 Data and methods

### 3.1 Data

We use two rounds of panel data on 1470 rural Indian women to study the impact of SHG membership on women’s empowerment. We also use data on 1344 rural Indian men (either the spouse of the respondent woman or another primary male decisionmaker within the household) to measure the impact of women’s membership in SHGs on men’s empowerment and the gender gap in empowerment. As mentioned earlier, the data used in this study was collected as part of the baseline and midline surveys of a larger four-year evaluation of the nutrition-intensification efforts led by PRADAN. PRADAN has worked to form and strengthen women’s SHGs and their higher-level federations since the early 1980s. It works primarily in marginalized communities, particularly those with high tribal populations. Along with SHG formation and capacity building it also engages women in livelihoods and agriculture to improve their economic empowerment and role in agriculture, and, in recent years, has expanded its thematic focus to include rights and entitlements, gender issues, and health, nutrition and sanitation. Along with PRADAN, several government-led State and National Rural Livelihoods Missions as well as NGOs also work to form and support SHGs in these same areas. As a result, women in our sample could be members of either PRADAN SHGs or those supported by these other organizations.

Our data are from eight districts in five states in eastern and central India (Madhya Pradesh, Chhattisgarh, Jharkhand, Odisha and West Bengal), determined in consultation with PRADAN. Three blocks were selected in each district of the study, from each block between five and seven villages were chosen at random from the full list of villages, and from each village, 20 women were selected at random from among all ever-married women aged 15–49. The final sample size at baseline was 2744 women from 136 villages in 24 blocks in 8 districts in 5 states. Sample selection was not conditioned on SHG membership, and at baseline approximately 38% of the female respondents in our sample belonged to an SHG. This number rose to 50% by midline; again, these SHGs are a mix of PRADAN and non-PRADAN SHGs.

The baseline survey was fielded from November 2015 to January 2016, and collected information on household socioeconomic and demographic characteristics, participation in SHG platforms and women’s empowerment, among other variables. The primary respondent was the woman, but some modules, including the WEAI, were also administered to a male member of the household (often the spouse of the respondent woman). At baseline, only 1674 out of 2744 (60%) of the households had both male and female respondents for the WEAI modules. Subsequent power calculations based on the overall evaluation design with three arms and eight clusters per arm determined the minimum required sample size to detect reasonable changes in WEAI indicators was 40% of the original baseline sample. During the midline survey, conducted from November 2017 to January 2018, we administered the WEAI-related modules to only that subsample of 1674 households for which we had baseline data on the WEAI-related modules from both a female and male respondent.^4^ This served to reduce both the cost of the survey and the burden on respondents. After accounting for attrition, we ended up with a subsample of 1470 women and 1344 men, which forms the sample for our analysis in this paper. Finally, since the pro-WEAI was still being developed when the midline survey was fielded, we implemented a partial pro-WEAI module that included only 10 of the 12 indicators. This should be considered when comparing our results to studies using the full pro-WEAI.

Table 2 presents descriptive statistics on characteristics of the respondent women, households and villages. Women in our sample are about 32–33 years old, 14 percent have 5 or fewer years of schooling and 26–29 percent do not work outside the home. SHG members are more likely to be older and have been married longer. Most women in the sample co-reside with their husbands, while slightly more than 20 percent (close to 15 percent) also co-reside with their mothers-in-law (fathers-in-law).

**Table 2:** Summary Statistics: Baseline Covariates by SHG membership at midline.

	SHG Membership at midline		Difference in Means
	Nonmembers (N = 755)	Members (N = 715)	Nonmembers vs Members
**Respondent woman characteristics**			
Age (years)	32.02	33.43	1.41**
			(0.52)
Age-squared	1,098.13	1,186.44	88.31**
			(36.09)
Has 1–5 years of schooling	0.14	0.14	0.004
			(0.018)
Has more than 5 years of schooling	0.21	0.20	−0.01
			(0.03)
Not working outside the home	0.293	0.26	−0.037
			(0.024)
Married	0.98	0.98	−0.005
			(0.008)
No. of years married	14.63	16.14	1.50**
			(0.56)

**Household characteristics**			
Time spent by women in the HH on fetching water from distant source, summer/winter	0.31	0.40	0.09*
			(0.05)
Husband lives in the household	0.97	0.97	−0.004
			(0.012)
Mother-in-law lives in the household	0.24	0.22	−0.02
			(0.02)
Father-in-law lives in the household	0.17	0.14	−0.03*
			(0.015)
Household size	4.87	4.89	0.02
			(0.09)
Caste of household head, SC	0.12	0.13	0.004
			(0.019)
Caste of household head, ST	0.69	0.61	−0.07*
			(0.04)
Caste of household head, OBC	0.15	0.20	0.05**
			(0.02)
Owns, rents/leases or uses/sharecrops land	0.89	0.92	0.02
			(0.02)
Ratio of male household members to female household members	1.23	1.28	0.05
			(0.05)
Dependency Ratio	0.82	0.88	0.07
			(0.045)

**Village characteristics**			
Average years of schooling among women in village	2.17	2.39	0.22
			(0.14)
Average land owned by HHs in village (acres)	1.97	1.87	−0.10
			(0.149)
Average no. of large livestock owned by HH in village	1.96	2.03	0.07
			(0.14)
Average no. of small livestock owned by HH in village	1.22	1.40	0.18
			(0.135)
Average Wealth Index among HHs in the village	−0.05	−0.04	0.01
			(0.14)
Caste of the HH head is the dominant caste in village	0.75	0.74	−0.003
			(0.023)
Village has at least one government primary school	0.87	0.90	0.04
			(0.03)
Village has at least one private primary school	0.07	0.08	0.01
			(0.02)
Village has at least one Anganwadi centre	0.88	0.90	0.02
			(0.025)
Village has electricity in all areas	0.80	0.82	0.029
			(0.030)
Distance to nearest town (in kms)	22.11	21.31	−0.803
			(2.181)

*Notes:* Standard errors in parenthesis. *, **, *** represent significance at 10, 5 and 1 percent, respectively.

The caste composition across SHG members and nonmembers is skewed with SHG members more likely to be OBC women and less likely to be ST. Village characteristics are not statistically significantly different for those individuals who are nonmembers versus those who are members, in other words, members and nonmembers belong to similar villages: Most villages have a government primary school and an Anganwadi (early childhood development) center, and are remote, located on average more than 21 km away from the nearest town.

Table 3 presents the overall empowerment indicators for the study sample and shows that more women than men are disempowered in this rural Indian sample, using either pro-WEAI or A-WEAI. We present two alternative empowerment scores for the pro-WEAI using different cut-offs. In the standard pro-WEAI case, based on 12 indicators, an individual is classified as empowered if they are empowered in 10 out of the 12 indicators. Because we only have 10 indicators, we compute the empowerment scores using two alternative cut-offs: (1) empowered in 7 out of 10 and (2) empowered in 8 out of 10 indicators. The aggregate empowerment score for women ranges between 0.66 and 0.73 using pro-WEAI (0.73 using A-WEAI); the men’s aggregate score for pro-WEAI ranges between 0.73 and 0.84 (0.83 for A-WEAI). The difference in the scores between the two measures is a result of the stricter threshold to be classified as empowered in pro-WEAI; indeed, the disempowered headcount (the percentage not achieving empowerment) is much higher for both women and men using pro-WEAI.

**Table 3:** Pro-WEAI and A-WEAI empowerment scores and gender parity.

**Indicator**	Pro-WEAI: Empowered if adequate in 7 out of 10 indicators		Pro-WEAIEmpowered if adequate in 8 out of 10 indicators		A-WEAI	
	**Women**	**Men**	**Women**	**Men**	**Women**	**Men**
N (number of observations)	1437	1339	1437	1339	1470	1343
**Empowerment score**	0.74	0.84	0.66	0.73	0.73	0.83
Disempowerment score	0.26	0.16	0.34	0.27	0.27	0.17
% achieving empowerment (empowered headcount)	0.45	0.64	0.19	0.29	0.36	0.54
% not achieving empowerment (disempowered headcount)	0.55	0.36	0.81	0.71	0.64	0.46
Mean empowerment score for not yet empowered (average adequacy score)	0.52	0.55	0.58	0.63	0.58	0.63
Mean disempowerment score for not yet empowered (average inadequacy score)	0.48	0.45	0.42	0.37	0.42	0.37
**Gender Parity Index (GPI)**	0.9		0.87		0.88	
N (number of dual-adult households)	1339		1339		1343	
% women achieving parity (1-HGPI)	0.59		0.51		0.53	
% women not achieving parity (HGPI)	0.41		0.49		0.47	
Average empowerment gap (IGPI)	0.26		0.26		0.25	
**Aggregate A-WEAI/Pro-WEAI score**	0.75		0.68		0.75	

The GPI, as described above, presents the proportion of households that achieve gender parity based on the intrahousehold inequality score; this is computed only for the subsample of dual adult households. Half the women in dual-adult households (51%) achieve gender parity using the pro-WEAI and the stricter threshold (8 indicators out of 10); the other half do not. These numbers are not substantially different for A-WEAI at 53% (47%) for those achieving (not achieving) gender parity. The percentage of women in dual adult households that achieve gender parity is 59% with the less strict cut-off of being empowered in at least 7 indicators.

**Fig. 1:**
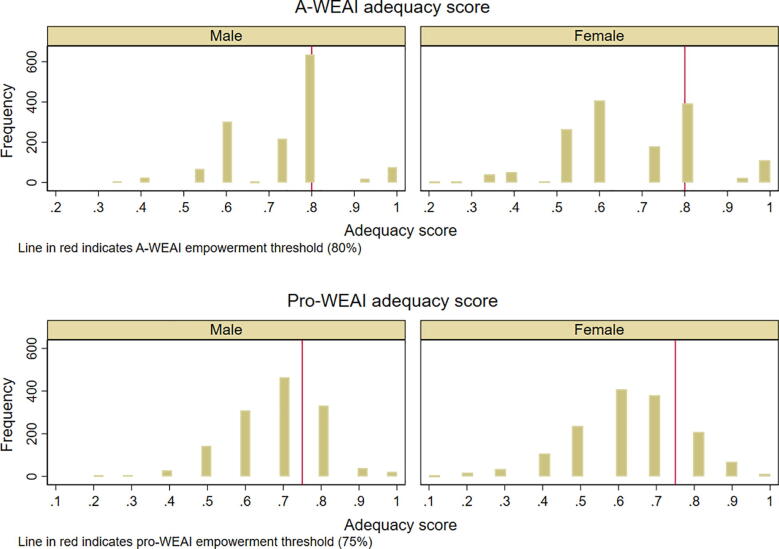
Combined adequacy score distribution.

The overall pro-WEAI score, a weighted average of the woman’s 3DE score and the GPI is 0.68 (for the 8 out of 10 cut-off) and 0.75 (for the 7 out of 10 cut-off); the corresponding value using A-WEAI is 0.75. A comparison of the distribution of pro-WEAI and A-WEAI shows that the distributions are fairly similar (Fig. 1). For the rest of the paper we use the stricter cut-off for the pro-WEAI empowerment scores.

Does the extent of disempowerment vary across households depending on whether the woman belongs to an SHG? Table 4 presents summary statistics for our outcome variables among the women and men in our sample, by women’s SHG membership status: (i) composite A-WEAI indicators: the 5DE score for the respondent woman, the measure of the gender gap between the respondent woman and her partner, (ii) for each of the component indicators, the underlying binary variables indicating whether the respondent woman/man is empowered in that indicator, and (iii) the continuous measures based on which the binary variables are constructed.

**Table 4:** Empowerment outcomes for women and men, members vs. nonmembers.

	Women's outcomes				Men's outcomes			
Empowerment outcome	N	Members	Non-Members	Difference in Means	N	Members	Non-Members	Difference in Means
**Aggregate score: pro-WEAI**								
Empowerment score (**3DE)**	1,437	0.715	0.607	0.107***	1,339	0.747	0.723	0.024**
Intrahousehold inequality score	1,337	0.059	0.092	−0.033***				

**Aggregate score: A-WEAI**								
Empowerment score (**5DE)**	1,470	0.785	0.682	0.103***	1,343	0.848	0.814	0.034***
Intrahousehold inequality score	1,343	0.072	0.107	−0.035***				

**Attitudes about intimate partner violence against women**								
0/1: Adequate in intimate partner violence against women domain	1,470	0.747	0.739	0.008	1,344	0.763	0.766	−0.003
Number of scenarios where respondent disagrees with IPV against women	1,470	4.341	4.415	−0.073	1,344	4.44	4.534	−0.094

**Respect among household members**								
Meets all 4 criteria for respect related to spouse, other respondent, or another household member	1,437	0.589	0.576	0.012	1,340	0.628	0.68	−0.052

**Instrumental agency, Input in productive decisions**								
0/1: Has input or feels can make decisions on all agricultural activities they participate in	1,470	0.824	0.763	0.061***	1,344	0.858	0.836	0.021
# activities for which respondent has some input or feels can make decisions	1,470	2.424	2.200	0.224**	1,344	2.499	2.440	0.059

**Ownership of land and other assets**								
0/1: Owns, solely or jointly, at least 3 assets, or land	1,470	0.966	0.954	0.013	1,344	0.988	0.973	0.015*
Total assets that are solely/jointly owned by respondent	1,470	6.478	6.207	0.272*	1,344	6.867	6.674	0.192*

**Access to and decisions on financial services**								
0/1: Adequate with respect to access to and decisions on financial services	1,470	0.786	0.703	0.083**	1,344	0.877	0.803	0.074**
# accessible sources where respondent solely/jointly participated in credit decisions	1,470	0.73	0.307	0.423***	1,344	0.55	0.365	0.185***

**Control over use of income**								
0/1: Has input in decisions on how to use income and output from all activities participated in	1,470	0.842	0.788	0.054*	1,344	0.917	0.877	0.040*
# activities respondent has input in decisions on income and output from all activities participated in	1,470	2.968	2.678	0.290**	1,344	3.354	3.249	0.105

**Work balance**								
0/1: Works <10.5 h/day (including time spent on childcare as a secondary activity)	1,470	0.354	0.374	−0.02	1,343	0.701	0.69	0.011
Number of hours worked = time spent on primary activity plus ½ time spent in childcare as secondary activity	1,470	11.096	10.988	0.108	1,343	8.906	8.921	−0.015

**Mobility**								
0/1: Adequate in mobility domain	1,470	0.951	0.968	−0.017	1,344	0.995	1	−0.005*
Number of places visited daily/weekly/biweekly/monthly or when required	1,470	4.099	3.793	0.306***	1,344	4.211	4.165	0.046

**Collective agency, Group membership**								
0/1 Active member of at least one group	1,470	0.439	0.024	0.415***	1,344	0.129	0.074	0.055***
No of groups where respondent is an active member	1,470	0.463	0.026	0.436***	1,344	0.145	0.08	0.066***

**Membership in influential groups**								
0/1: Active member of influential group	1,470	0.173	0.012	0.162***	1,344	0.058	0.032	0.026*
No. of influential groups respondent is a member of	1,470	0.186	0.013	0.173***	1,344	0.064	0.036	0.028**

***Notes:*** Standard errors in parenthesis. *, **, *** represent significance at 10, 5 and 1 percent, respectively.

Many of the women’s empowerment indicators presented in Table 4 are significantly better for SHG members compared to nonmembers – indicating that on average SHG members are more empowered. Overall empowerment scores are higher for SHG members, and empowerment gaps with men within the same household are significantly lower, using pro-WEAI and A-WEAI-based measures. Women who are SHG members have more input into agricultural production and decisions on financial services, make more decisions regarding financial services, and have greater input in income decisions. They are also likely to be active in more groups within their communities and to visit more places than non-SHG members, both of which may occur because of SHG-related activities. Although there appears to be a small and weakly significant difference in asset ownership between SHG members and nonmembers, we do not find any difference in terms of workload. Also, there is a lack of apparent difference in attitudes towards intimate partner violence and respect within the household.

Interestingly, men whose wives are SHG members are more empowered in several dimensions compared to those whose wives are nonmembers. Overall empowerment scores are higher for husbands of members, along with the number of assets solely or jointly owned, the number of credit sources in which the husband participates in decisionmaking (whether solely or jointly), group membership, and membership in influential groups. The only indicator on which husbands of SHG members do worse than those of nonmembers is mobility, but this is only weakly significant, and the magnitude of the impact is small. In addition, this finding is consistent with the higher number of places that SHG member women visit; if wives are able to visit these places more freely, they may not need their husbands to.

Although differences in empowerment indicators may be attributable to SHG membership, these descriptive statistics do not account for intrinsic or extrinsic differences between women who choose to be SHG members and those that do not, and their husbands. We address this selection bias in the empirical strategy below.

### 3.2 Empirical strategy

To assess the impact of SHG membership on women’s empowerment, one needs to consider that access to SHG membership in our sample was not random, and most villages in our sample already had some SHG presence during the baseline survey. Membership is voluntary, and women self-select to be SHG members. The primary identification challenge is to isolate the causal link between SHG membership and our measures of empowerment from any effects of omitted or unobservable factors that may be driving the outcome of interest and be correlated with SHG membership.

In a simple ordinary least squares (OLS) specification as given in equation 1 below, we would regress our measure of women’s empowerment for individual iat time t, $Y_{\mathrm{it}}$, on an indicator for SHG membership, $\mathrm{SHGmembe}r_{\mathrm{it}}$. In this specification, $\beta_{1}$is the coefficient of interest. However, in the absence of random assignment of women to SHGs, i.e. where $cov\left(SHGmember_{\mathrm{it}},\varepsilon_{\mathrm{it}}\right)\neq 0$, $\beta_{1}$ is likely to be a biased estimate of the effect of SHG membership on women’s empowerment.

$$Y_{\mathrm{it}}=\beta_{0}+\beta_{1}SHGmember_{\mathrm{it}}+\varepsilon_{\mathrm{it}}$$

OLS estimates are potentially biased if women who decide to join an SHG are fundamentally different from those who do not. Self-selection into SHG membership could confound the effect of the unobservable differences between members and nonmembers with the effect of belonging to an SHG, thereby biasing the impact estimate of SHG membership. In our study, the lack of a clear geographical placement and/or targeting criteria and SHGs’ presence in these areas prior to our baseline survey precluded the use of a randomized controlled trial or regression discontinuity design as an identification strategy. We therefore decided to apply matching methods to examine the impact of SHG membership on women’s empowerment.

We construct a comparison group by matching SHG members to nonmembers based on observable respondent, household and community characteristics. We estimate impacts of SHG membership using nearest neighbor matching (NNM), a form of covariate matching in which the comparison group sample of nonmembers is selected based on similarity to the SHG member sample on observable characteristics (Abadie et al., 2004, Abadie and Imbens, 2006)^5^. NNM matches members and nonmembers to minimize the average difference in characteristics, using a multidimensional metric to determine the weights for constructing the average. The effect of being an SHG member is then measured as the average difference in the outcome for each SHG member from the average outcome among its matched nonmembers. We matched using five nonmembers as neighbors. We also use the bias correction method proposed by Abadie and Imbens (2006) because we match on more than two continuous variables.^6^

Some details and limitations of the matching procedures used deserve attention. Matching is based on variables that are associated both with the probability of being an SHG member and with the outcome of interest (Heckman & Navarro-Lozano, 2004). However, these variables should be determined before the SHGs were established to ensure that they were not affected by SHG membership itself. Given that SHGs existed in our sample villages at baseline, we do not have data on these observables before the women became members. To reduce the risk of endogeneity bias, we use SHG membership at midline as our membership variable and match on variables measured at baseline that were predetermined and exogenous (to SHG membership). The matching variables can be grouped in five categories: respondent women’s characteristics, household characteristics, including time spent by women in the household on fetching water^7^, village characteristics, district and state effects. The full list of covariates used for matching is found in Table 2.

To aid comparison and examine the nature and direction of the bias, we also present the results from the OLS estimations using the same sets of outcomes and the matching variables as covariates. The estimating equation is as follows:

$$Y_{\mathrm{ihvdst}}=\alpha +\beta {\mathrm{SHGmember}}_{\mathrm{ihvdst}}+\gamma W_{\mathrm{ihvdst}-1}+\theta X_{\mathrm{hvdst}-1}+\pi C_{\mathrm{vdst}-1}+\delta_{d}+\varepsilon_{\mathrm{ihvdst}}$$

where $Y_{\mathrm{ihvdst}}$ is the outcome of interest for woman *i* in household *h* in village *v* in district *d* of state *s* at time t (midline), $\mathrm{SH}{\mathrm{Gmember}}_{\mathrm{ihvdst}}$ is a dummy variable indicating whether the respondent woman is an SHG member, $W_{\mathrm{ihvdst}-1}$ is the vector of the respondent woman’s characteristics at time t-1(baseline) mentioned above,$X_{hvdst\_-1}$ and $C_{\mathrm{vdst}-1}$ are vectors of baseline household and village characteristics, respectively; and $\delta_{d}$ are district dummies.^8^ Finally, $\varepsilon_{\mathrm{ihvdst}}$ is the individual-specific error term clustered at the block level.

### 3.3 Ethical approval and study registration

Ethical approval was sought from a local institutional review board in India as well as from the [ Anonymized for peer review]. Prior to baseline we registered the larger evaluation study at 3ie’s Registry for International Development Impact Evaluations with the study ID: RIDIE-STUDY-ID-xxxxx [registration number not revealed for peer review].

## 4 Results

In this section we first examine the impact of SHG membership on the aggregate empowerment measures - the empowerment scores (3DE and 5DE) and the intrahousehold inequality score which represents the gender gap in empowerment within the same household, and then unpack these results by examining the component indicators in both their binary and continuous forms. Because adequacy is assessed using uniform thresholds or cutoffs, binary indicators may not be as sensitive to changes as the continuous indicators. Wherever relevant, impact estimates are presented for both women and men. In the case of men, comparisons are made between men whose partners are SHG members and men whose partners do not belong to an SHG. All impact estimates discussed in the text refer to the NNM results, unless otherwise mentioned.

### 4.1 Women’s empowerment score and the empowerment gap

Using both pro-WEAI and A-WEAI, we find significant impacts of SHG membership on the women’s empowerment score and the intrahousehold inequality score (Table 5). Being an SHG member increases the overall female empowerment score by 10.2 percentage points (pp) (9.8 pp using A-WEAI), which is 16.8 percent over the average empowerment score among nonmembers (14.4 percent using A-WEAI), p < 0.01. SHG membership also reduces the gap between male and female empowerment scores within a household by 3.1 pp (3.7 pp using A-WEAI), which translates to a reduction of 33.7 percent (34.6 percent) over the average among the nonmembers, p < 0.01. These are large and appreciable differences in women’s empowerment and the intrahousehold inequality score because of SHG membership. It is important to note that at the aggregate level both indices – pro-WEAI and A-WEAI – show similar results, which is unsurprising given their similar distributions.

**Table 5:** Effect of SHG membership on women's and men's empowerment scores and the intrahousehold inequality score, nearest neighbor matching estimates.

Outcome			**Women**			**Men**		
			**OLS**	**NNM**	**Control group mean**	**OLS**	**NNM**	**Control group mean**
**Pro-WEAI (8 of 10)**								
Empowerment score (3DE)			0.097***	0.102***	0.607	0.016	0.015	0.723
Women: N = 1434			(0.011)	(0.011)		(0.012)	(0.012)	
Men: N = 1337								
Intrahousehold inequality score			−0.033***	−0.031***	0.092			
Based on dual-adult household sample, N = 1335			(0.011)	(0.009)				

**A-WEAI**								
Empowerment score (5DE)			0.088***	0.098***	0.682	0.025**	0.020*	0.814
Women: N = 1467			(0.018)	(0.012)		(0.011)	(0.012)	
Men: N = 1341								
Intrahousehold inequality score			−0.034**	−0.037***	0.107			
Based on dual-adult household sample, N = 1341			(0.012)	(0.008)				

Notes: Standard errors in parenthesis. *, **, *** represent significance at 10, 5 and 1 percent, respectively.

In addition, we find a marginally significant and positive impact on the empowerment score among husbands of SHG members compared to husbands of women who are not SHG members using A-WEAI. Although the point estimate is similar for pro-WEAI and A-WEAI, only the latter is statistically significant. These aggregate findings suggest that women’s membership in SHGs can empower them, reduce the empowerment gap and to some extent also empower their husbands. In other words, we can safely say that membership in SHGs can empower women without disempowering the men within their household.

### 4.2 Impacts on empowerment indicators

#### 4.2.1 pro-WEAI indicators

Impact estimates using the aggregate measures show that SHG membership significantly and meaningfully improves overall empowerment. However, these composite measures do not tell us which dimensions of empowerment are improving relative to others. To understand possible pathways of impact, we examine the pro-WEAI domains and indicators in Table 6, and the A-WEAI domains and indicators in Table 7, discussing both the binary indicator and the continuous indicator from which it is derived.

**Table 6:** Effect of SHG membership on pro-WEAI domains and component indicators.

	**Women**			**Men**		
	**OLS**	**NNM**	**Control group mean**	**OLS**	**NNM**	**Control group mean**
**Intrinsic agency**						
Attitudes about intimate partner violence against women	N = 1467			N = 1342		
0/1: Adequate in intimate partner violence against women domain	0.005	−0.010	0.739	0.008	0.009	0.766
	(0.022)	(0.026)		(0.024)	(0.0260)	
Number of scenarios where respondent disagrees with IPV against women	−0.072	−0.088	4.415	−0.063	−0.0183	4.534
	(0.056)	(0.08)		(0.063)	(0.0711)	

Respect among household members	N = 1434			N = 1338		
Meets all 4 criteria for respect related to spouse, other respondent, or another household member	0.012	0.032	0.576	−0.064*	−0.036	0.68
	(0.021)	(0.030)		(0.035)	(0.031)	

**Instrumental agency**						
Input in productive decisions	N = 1,467			N = 1,342		
0/1: Has input or feels can make decisions on all agricultural activities they participate in	0.053**	0.035	0.763	0.029	0.017	0.836
	(0.021)	(0.024)		(0.022)	(0.022)	
# activities for which respondent has some input or feels can make decisions	0.173*	0.220***	2.200	0.032	0.029	2.440
	(0.100)	(0.085)		(0.084)	(0.074)	

Ownership of land and other assets	N = 1467			N = 1342		
0/1: Owns, solely or jointly, at least 3 assets, or land	0.01	0.009	0.954	0.013	0.006	0.973
	(0.012)	(0.012)		(0.009)	(0.008)	
Total assets that are solely/jointly owned by respondent	0.168	0.224*	6.207	0.105	0.095	6.674
	(0.110)	0.121		(0.096)	(0.109)	

Access to and decisions on financial services	N = 1,467			N = 1,342		
0/1: Adequate with respect to access to and decisions on financial services	0.080**	0.094***	0.703	0.063**	0.0428*	0.803
	(0.034)	(0.028)		(0.024)	(0.023)	
# accessible sources where respondent solely/jointly participated in credit decisions	0.388***	0.395***	0.307	0.211***	0.149***	0.365
	(0.063)	(0.052)		(0.067)	(0.053)	

Control over use of income	N = 1467			N = 1342		
0/1: Has input in decisions on how to use income and output from all activities participated in	0.034	0.019	0.788	0.04**	0.04**	0.877
	(0.024)	(0.024)		(0.018)	(0.021)	
# activities respondent has input in decisions on income and output from all activities participated in	0.206*	0.275***	2.678	0.063	0.088	3.249
	(0.109)	(0.102)		(0.085)	(0.085)	

Work balance	N = 1467			N = 1341		
0/1: Works<10.5 h/day (including time spent on childcare as a secondary activity)	−0.046	−0.024	0.374	−0.002	0.010	0.690
	(0.027)	(0.030)		(0.019)	(0.028)	
Number of hours worked = time spent on primary activity plus ½ time spent in childcare as secondary activity	0.288	0.070	10.99	0.095	−0.039	8.921
	(0.185)	(0.165)		(0.192)	(0.191)	

Mobility	N = 1467			N = 1342		
0/1: Adequate in mobility domain	−0.01	−0.008	0.968	−0.006	−0.005*	1.000
	(0.013)	(0.011)		(0.003)	(0.003)	
Number of places visited daily/weekly/biweekly/monthly or when required	0.305***	0.299***	3.793	0.024	0.012	4.165
	(0.069)	(0.062)		(0.037)	(0.055)	

**Collective agency**						
Group membership	N = 1467			N = 1342		
0/1 Active member of at least one group	0.396***	0.411***	0.024	0.024	0.028	0.074
	(0.039)	0.02		(0.018)	(0.02)	
No of groups where respondent is an active member	0.413***	0.432***	0.027	0.034	0.042*	0.08
	(0.04)	(0.022)		(0.021)	(0.022)	

Membership in influential groups	N = 1467			N = 1342		
0/1: Active member of influential group	0.143***	0.160***	0.012	0.016	0.022	0.032
	(0.019)	(0.015)		(0.012)	(0.014)	
No. of influential groups respondent is a member of	0.151***	0.170***	0.013	0.017	0.025	0.036
	(0.02)	(0.017)		(0.014)	(0.015)	

*Notes:* Standard errors in parenthesis. *, **, *** represent significance at 10, 5 and 1 percent, respectively.

**Table 7:** Effect of SHG membership on the A-WEAI domains and component indicators.

	**Women**			**Men**		
	**OLS**	**NNM**	**Control group mean**	**OLS**	**NNM**	**Control group mean**
**Production domain**	N = 1467			N = 1342		
0/1: Input in decisions in at least 2 domains	0.049**	0.034*	0.833	0.007	0.003	0.918
	(0.021)	(0.020)		(0.020)	(0.015)	
# activities for which respondent has some input or feels can make decisions	0.173*	0.220***	2.200	0.032	0.029	2.440
	(0.100)	(0.085)		(0.084)	(0.074)	

**Resources domain**	N = 1467			N = 1342		
Assets						
0/1: Solely/jointly owns at least two small assets or one large asset (including land)	0.005	0.011**	0.992	0.001	0.001	0.999
	(0.005)	(0.004)		(0.001)	(0.001)	
Total assets that are solely/ jointly owned by respondent	0.168	0.224*	6.207	0.105	0.095	6.674
	(0.11)	(0.121)		(0.096)	(0.109)	

Credit						
0/1: Adequate in the credit domain	0.079**	0.089***	0.605	0.068***	0.049**	0.734
	(0.03)	(0.029)		(0.024)	(0.024)	
# accessible sources where respondent solely/jointly participated in credit decisions	0.388***	0.395***	0.307	0.211***	0.149***	0.365
	(0.063)	(0.052)		(0.067)	(0.053)	

**Income domain**	N = 1467			N = 1342		
0/1: Input in income decisions in at least one domain^a^	0.004	0.001	0.995			1.000
	(0.002)	(0.001)				
No. of domains individual has input in/feels can make decisions	0.243**	0.242**	5.220	−0.003	0.040	6.055
	(0.105)	(0.096)		(0.08)	(0.082)	

**Leadership domain**	N = 1467			N = 1342		
0/1: Active member of at least one group	0.396***	0.411***	0.024	0.024	0.028	0.074
	(0.039)	(0.02)		(0.018)	(0.02)	
No. of groups where respondent is an active member	0.413***	0.432***	0.027	0.034	0.042*	0.08
	(0.04)	(0.022)		(0.021)	(0.022)	

**Time domain**	N = 1467			N = 1341		
0/1: Works<10.5 h per day (not including time spent on childcare as a secondary activity)	−0.080**	−0.053*	0.450	0.009	0.014	0.722
	(0.031)	(0.03)		(0.017)	(0.028)	
Total hours worked (not including time spent on childcare as a secondary activity)	0.353*	0.192	10.40	0.034	−0.0496	8.617
	(0.19)	(0.16)		(0.151)	(0.177)	

*Notes:* Standard errors in parenthesis. *, **, *** represent significance at 10, 5 and 1 percent, respectively. a. All men in the sample have some input in income decisions so we were unable to estimate an impact of their partners’ SHG membership on the income indicator.

Pro-WEAI has four indicators in the intrinsic agency domain, of which two were collected in this study.^9^ SHG membership does not appear to significantly impact attitudes towards intimate partner violence against women nor respect among household members for both women and men, irrespective of whether binary or continuous indicators are used. The lack of a statistically significant impact on attitudes towards intimate partner violence is in line with the null effects on similar outcomes reported in Brody et al. (2017). This is not entirely surprising - not only are these social norms harder to move over a short period of time, the set of interventions we study did not provide direct transfers but focused instead on information provision and behavior change communication (BCC). There is evidence that cash and in-kind transfers to women can help reduce intimate partner violence, but these effects are typically not sustained beyond the program period (Buller et al., 2016, Buller et al., 2018). Roy, Hidrobo, Hoddinott, and Ahmed (2018) use data from Bangladesh to show that transfers combined with group-based intensive BCC significantly reduce the incidence of intimate partner violence even six to ten months after the program had ended, with BCC being the key intervention component driving the observed effects. However, our at-scale programmatic setting did not match either the frequency or intensity of the BCC sessions in Roy et al. (2018) closely controlled experimental set-up.

The instrumental agency domain has six indicators. We are unable to detect significant impacts of SHG membership on the binary indicators for women, with the exception of adequacy with respect to access to and decisions on financial services. The continuous indicators, which are not sensitive to the choice of adequacy cutoffs, provide a more nuanced picture. SHG membership increases the number of agricultural activities for which the woman has some input or feels that she can make decisions (10%, p < 0.01), the number of assets solely or jointly owned by the respondent (3.6%, p < 0.10), the number of accessible sources where the respondent woman solely or jointly participated in credit decisions (128%, p < 0.01), the number of activities in which she has input on income decisions (10.2%, p < 0.01), and the number of places that she can visit when required (7.9%, p < 0.01). The need to attend SHG and higher-level federation meetings of these groups could underlie the significantly greater mobility among SHG members. In addition, we find that having women who are SHG members led to an increase in men’s input into decisions in using income and agricultural output and over credit but reduces their adequacy in the mobility domain. The weakly negative impact on men’s adequacy with respect to mobility may indicate men’s reducing visits to locations that women themselves are now able to visit. Improved decisionmaking for men in the credit domain could reflect the spillover impacts of greater financial literacy and empowerment that SHG women acquire through the group savings and credit activities. Improved male decisionmaking over the use of income could be a result of higher overall agricultural income as a results of SHG-based extension activities, with higher stakes encouraging the men to exert more control over the use of this income.

SHG membership also positively affects the two indicators of **collective agency** for women, being an active member of at least one group and membership in an influential group. Impacts on both binary and continuous collective agency indicators are positive and significant for women but not for men. Since our ‘treatment’ is membership in an SHG, it follows that ‘treated’ women (those who are SHG members) are more likely to fulfil the adequacy criteria in this domain than women in the comparison arm (those who are not SHG members). We do allow for a broad range of possible groups in the questionnaire, of which SHGs are just one variant, however, the very low control group mean of only 0.03 groups where women are active members suggests that the predominant form of group within the community is indeed the SHG. Given this, the reader should interpret this impact estimate as being largely mechanical.

#### 4.2.2 A-WEAI estimates

Estimates using A-WEAI are organized according to the five domains of empowerment and are presented in Table 7. Once again, we estimate impacts on both binary and continuous indicators.

A person is considered adequate in the production domain if he/she has input in production decisions in at least two sub-domains. Being an SHG member only has a weakly significant impact on this binary indicator for women, but no impact for men (Table 7). For women, SHG membership has a significant impact on the number of agricultural sub-domains in which the individual has some input in making decisions or feels that she can make them (p < 0.01); there is no corresponding impact on men whose partners are SHG members. This is consistent with earlier results from Raghunathan et al. (2019) documenting increases in women’s participation in some agricultural decisions.

The resource domain is composed of two binary indicators, one of which is related to sole or joint ownership of at least two small assets or one large asset, and the second which measures access to and participation in credit decisions. NNM estimates show that SHG membership significantly increases the probability of a woman owning at least two small assets or one large asset by 1 pp (p < 0.05) and has a positive effect on the total number of assets solely or jointly owned. There is no effect of SHG membership on men’s asset ownership. SHG membership has a significant impact on women’s and men’s adequacy in the credit sub-domain, 9 pp (p < 0.01) in the case of women and 5 pp (p < 0.05) in the case of men. The number of accessible credit sources for which the woman participates in decisionmaking is higher by 0.39 (p < 0.01) among SHG members as compared to nonmembers, representing a dramatic 128% of the average number of accessible credit sources for nonmembers. This result is not surprising, given the strong emphasis of SHGs on financial inclusion through their savings and credit activities. Perhaps more surprisingly, the number of credit sources for which the man participates in decisionmaking is higher among husbands of SHG members by 0.15, which is a sizeable 41 percent of the corresponding average among nonmembers.

An individual is considered adequate in the income domain if he/she has input in income decisions in at least one sub-domain. SHG membership does not affect the probability that a woman has input in decisions in at least one income domain but increases the number of domains in which she has some input in decisions by 0.24 (p < 0.05). This is 4.6% higher than the corresponding average for the control group. All men in the sample have some input in income decisions so we were unable to estimate an impact of their partners’ SHG membership on the income indicator. We see no impact on the number of domains where the man has input or feels that he can make decisions.

As mentioned above, since SHGs are, by definition, groups, it follows that women in the treatment arm satisfy the adequacy criteria in the leadership domain, which is defined as being an active member of at least one group. SHG membership increases the probability that the respondent is an active member of at least one group by 41 pp (p < 0.01) and increases the number of groups of which the respondent is an active member by 0.43 (p < 0.01).^10^ While we expect this positive impact on women’s groups owing to the design of the intervention, we do see a small but significant increase of 0.04 in the number of groups where the male respondent is an active member; this is a 52.5% increase over the control group mean of 0.08.

Finally, SHG membership causes an increase in workload – reducing the likelihood of working <10.5 h a day by 5.3 pp (about 12 percent over the control group mean, p < 0.1). However, there is no significant impact on the continuous workload indicator in the NNM estimate, even though the OLS estimate shows a weakly significant increase of 0.35 h or 20 min spent working per day, where working hours are defined as the total number of hours spent on market and nonmarket work (including time spent on domestic work and caring for children and sick and elderly). There is no impact of women’s SHG membership on the number of hours worked by the male respondents. In general, men work about two hours less per day than women, regardless of their wives’ membership status, although workload is also a major contributor to men’s disempowerment.

Overall, we find that the OLS estimates and the NNM estimates are not very different from each other. This indicates the homogeneity of the sample – women in our sample are very alike on observable characteristics whether or not they are SHG members.

#### 4.2.3 Robustness to exclusion of SHG membership from the empowerment indicators

We have noted previously that group membership is one of the component indicators of the women’s empowerment index. Though we ask about a range of possible groups, the predominant form of group membership among individuals in our sample is membership in an SHG. As a result, one might argue that the observed impact of SHG membership on empowerment in the domain of group membership or collective agency is by construction. To check for this, we re-estimate the models for all empowerment indicators – the composite 5DE (A-WEAI)/3DE (pro-WEAI) scores and intrahousehold inequality score, and the underlying sub-domain binary and continuous indicators - by excluding SHG membership from the group membership indicator.

As would be expected, this modification alters our results on the composite measures and group membership indicators among women (Table 8, Table 9)^11^. Once we exclude SHG membership from the group membership indicator, the impact estimate on the aggregate pro-WEAI empowerment score (3DE) is much smaller in magnitude but remains statistically significant (0.020, p < 0.05; Table 8). The coefficients on other composite indicators - intrahousehold inequality score for pro-WEAI and A-WEAI and the 5DE score for A-WEAI - are smaller in magnitude and lose statistical significance. There is no change in the corresponding empowerment scores for the men. Table 9 presents the impact estimates for the collective agency (pro-WEAI) and leadership (A-WEAI), here too the coefficients are smaller, but remain statistically significant. These results show that SHG membership is an important factor contributing to the improvement in women’s empowerment and the reduction in the intrahousehold inequality score.

**Table 8:** Effect of SHG membership on women's and men's empowerment scores and the intrahousehold inequality score when SHG membership is excluded from the group membership indicator, nearest neighbor matching estimates.

Outcome			**Women**		
			**OLS**	**NNM**	**Control group mean**
**Pro-WEAI (8 of 10)**					
Empowerment score (3DE)			0.020**	0.021**	0.607
Women: N = 1434			(0.007)	(0.010)	
Men: N = 1337					
Intrahousehold inequality score			−0.006	−0.003	0.092
Based on dual-adult household sample, N = 1335			(0.010)	(0.010)	

**A-WEAI**					
Empowerment score (5DE)			0.004	0.012	0.682
Women: N = 1467			(0.012)	(0.012)	
Men: N = 1341					
Intrahousehold inequality score			0.002	−0.001	0.107
Based on dual-adult household sample, N = 1341			(0.011)	(0.009)	

*Notes:* Standard errors in parenthesis. *, **, *** represent significance at 10, 5 and 1 percent, respectively.

**Table 9:** Effect of SHG membership on collective agency and leadership domains and component indicators when SHG membership is excluded from the group membership indicator.

	**Women**		
**Pro-WEAI**	**OLS**	**NNM**	**Control group mean**
**Collective agency**			
Group membership	N = 1467		
0/1 Active member of at least one group	0.022**	0.022**	0.024
	(0.010)	(0.009)	
No of groups where respondent is an active member	0.022**	0.024**	0.027
	(0.010)	(0.011)	
Membership in influential groups	N = 1467		
0/1: Active member of influential group	0.015**	0.015**	0.012
	(0.006)	(0.008)	
No. of influential groups respondent is a member of	0.014*	0.014	0.013
	(0.008)	(0.009)	

**A-WEAI**	**OLS**	**NNM**	**Control group mean**
**Leadership domain**	N = 1467	N = 1342	
0/1: Active member of at least one group	0.022**	0.022**	0.024
	(0.010)	(0.009)	
No. of groups where respondent is an active member	0.022**	0.024**	0.027
	(0.010)	(0.011)	

*Notes:* Standard errors in parenthesis. *, **, *** represent significance at 10, 5 and 1 percent, respectively.

## 5 Discussion and conclusion

This paper aimed to assess the impact of women’s SHG membership on various aspects of their empowerment, measured using the pro-WEAI and A-WEAI. In addition to measuring the aggregate impact of SHG membership on empowerment and on the intrahousehold inequality score, the decomposability of the WEAI family of empowerment indicators allows us to identify specific domains of empowerment that are most affected by SHG membership. This not only helps us understand which pathways between SHG membership and empowerment are relevant in the Indian context, but also allows us to identify possible tradeoffs and unintended consequences of participation in SHGs on women’s well-being. Methodologically, comparing the results from these two WEAI variations, enables us to assess the suitability of these indices for assessing empowerment impacts at the project level.

Our results show that SHG participation has significant and quantitatively meaningful impacts on the overall empowerment score as well as the empowerment gap between spouses (both pro-WEAI and A-WEAI), and a weak positive impact on the overall empowerment scores for men (only A-WEAI). We can thus infer that the emphasis on women’s empowerment in SHG programming has been effective overall. Importantly, we also find that closing the intrahousehold empowerment gap does not appear to have been a result of disempowering the men in the household, a potential unintended consequence of programs aimed solely at women.

In terms of pathways of impact, we observe strong positive impacts on women’s credit access and decision making as well as control over income use. This is not surprising, given that SHGs are platforms that are primarily used for facilitating savings and credit activities among group members. At the same time, there are areas where SHG participation appears to have weaker impacts, such as women’s decisionmaking on production and asset ownership, areas that embody deep-seated gender norms about women’s participation in agriculture and women’s asset ownership, norms that are slower and harder to change.

SHG membership mechanically improves outcomes related to the leadership domain, which, in the WEAI context, refers to active participation in groups. What is surprising is that the participation of women in these groups also seems to have an impact on men’s group participation. Along with the financial benefits of belonging to an SHG, the network effects of these groups are empowering in themselves, especially for rural women with limited social ties and access to information. The specific agriculture and livelihoods related information and trainings provided in the group meetings could increase women’s confidence in participating in decision-making in their own homes. For example, a cash transfer program in rural Bangladesh, complemented with nutrition behavior change communication and trainings provided in a group setting to women, had long term impacts on reducing intimate partner violence and improvements in women’s empowerment (Roy et al., 2018). This example shows that an intervention delivered through a group-based platform had impacts on intrahousehold relationships, even when the messaging was not tailored to women’s empowerment. The financial literacy and comfort in dealing with money and transactions that women gain from participating in the group’s savings and credit activities could enable them to take greater control over household resources.

We have noted elsewhere that since SHG membership in our data is neither randomly allocated nor amenable to a regression continuity design, we identify the effect of SHG membership using nearest neighbor matching. We acknowledge the limitation that matching is based on observable characteristics and therefore cannot fully account for all risks of bias. However, similar criticisms could also apply to other methods, for example, in a randomized controlled trial where the control arm may experience spillovers and thus be contaminated. We believe that the matching model provides plausible causal estimates in this real-life programmatic context.

In terms of advancing the methodology for assessing empowerment impacts, in aggregate, both the A-WEAI and pro-WEAI indices yield qualitatively similar results owing to the similarity of their distributions. However, the differences in index construction result in slightly different results for some indicators. For example, we find a weak positive impact on men’s empowerment score measured using 5DE but not using 3DE. The impact on the 5DE seems to be largely driven by the impact on credit related indicators for men in the A-WEAI. The pro-WEAI indicators also show a similar impact but the weighting in pro-WEAI, which involves higher cutoffs, is likely the reason behind no aggregate impact. More importantly, pro-WEAI captures intrinsic agency, an important aspect of empowerment, for which we are unable to detect any impact. We would not have detected this absence of impact using A-WEAI, which does not include the intrinsic agency indicators. The theory of change underlying the intervention may serve as a guide in making the appropriate choice between the two index variations; programs that emphasize changes in gender norms surrounding intimate partner violence and intrahousehold relationships may want to use the more finely-tuned pro-WEAI for project impact assessment. Our findings also underscore the importance of qualitative research on the perceptions of empowerment among women and men; such qualitative research can provide greater insight on the different factors that increase empowerment and those that limit it. Better understanding of these constructs are crucial to furthering their measurement.

A few commonly-raised concerns deserve mention here. First, there is criticism that the SHG platform imposes a significant cost on women’s time. The additional costs women incur as a result of participating in time-intensive interventions has also been noted in other contexts, where engagement in such interventions might compete directly with caregiving or time spent preparing food (Carlson et al., 2015, Johnston et al., 2015, Komatsu et al., 2018), though evidence in the microcredit context is limited (Garikipati, 2012). We find some evidence to support that concern using A-WEAI, with our results showing that SHG participation increases workload, albeit by a small and only marginally significant amount. Being an SHG member may involve additional responsibilities that add to a women’s workload, such as attending meetings, engaging in collective action, or undertaking livelihoods activities.

Second, whether SHGs are inclusive and whether they reach women from marginalized groups are valid concerns. We noted earlier that the NGO PRADAN deliberately focuses on marginalized communities in the areas where it works, as reflected in the high proportion of Scheduled Caste and Tribe members in our sample. However, as noted by Nichols (2020) in a similar SHG-based agriculture-nutrition intervention in India, there are several intersecting factors that affect women’s ability and desire to participate actively in SHG-led programs. Poorer women and those who are younger, have small children, belong to smaller households with less labor and hence potentially face a greater opportunity cost of time are all less likely to participate in SHGs and SHG-led agricultural or other programs. The exclusion of the poorest of the poor from participation in SHG programs is also noted in Brody et al. (2017). NGOs organizing these groups are themselves constrained by limited budgets, reporting requirements to donors (which often focus on quantitative metrics of reach and uptake), and by the amount of time and effort required to train volunteers and frontline workers to target marginalized groups, particularly when that marginalization is linked also to geographic location and language. While we do not find large significant differences in our data between SHG members and non-members, and while our matching exercise controls for this issue to some extent, it is worth noting that participation in these programs is not always equitable.

Third, the question of whether SHGs can be expected to change deeply entrenched social norms, such as those regarding gender roles, has also been raised by researchers and implementors. We show that while SHG membership has a small but highly significant impact on the number of places women can go, a measure of mobility, it does not affect other indicators of empowerment such as attitudes towards intimate partner violence, and trust and intrahousehold harmony. These reflect more entrenched gender norms that are often reinforced by community expectations and are hard to change at an individual level or through any single intervention. The limited impact on the production domain and the intrinsic agency indicators of intrahousehold harmony and attitudes towards intimate partner violence suggests that gender norms are slow to change, and that SHG programming may need to deliberately address changing these norms by reaching out more directly to other members of the community. There is a growing consensus on the need to change social norms and psychosocial determinants of women’s empowerment and participation, and; some evidence on the ways to bring about this change (Fiala, 2018, Siba, 2019). We need to simultaneously acknowledge that SHGs alone might not be able to do so, especially since they engage only one side of the gender equation (de Hoop et al., 2014, Jakimow and Kilby, 2006). However, measuring gender norms is a key first step to diagnosing and solving this problem, which is why the inclusion of these indicators in pro-WEAI is welcome. In addition, for gender norms to change, SHG programs also need to target husbands, in-laws, and community leaders. Pilot programs such as the Agriculture, Nutrition, and Gender Linkages (ANGeL) program in Bangladesh, discussed in Quisumbing, Ahmed, Hoddinott, Pereira, & Roy (2020), show the potential of gender sensitization efforts that reach both women and men in changing attitudes towards gender. By using indicators that are sensitive to changes in intrinsic agency, programs will be better equipped to assess whether they have achieved their empowerment objectives.

## CRediT authorship contribution statement

**Neha Kumar:** Conceptualization, Methodology, Writing - original draft, Writing - review & editing, Supervision, Funding acquisition. **Kalyani Raghunathan:** Conceptualization, Methodology, Writing - original draft, Writing - review & editing. **Alejandra Arrieta:** Formal analysis, Writing - original draft, Writing - review & editing. **Amir Jilani:** Formal analysis, Writing - original draft, Writing - review & editing. **Shinjini Pandey:** Formal analysis, Writing - original draft, Writing - review & editing.

## Declaration of Competing Interest

The authors declare that they have no known competing financial interests or personal relationships that could have appeared to influence the work reported in this paper.

## Figures and Tables

**Table:** 

	**WEAI sub-domain**					
	**Production**	**Assets**	**Credit**	**Income**	**Group membership**	**Time allocation**
Detectable difference for women	0.11	0.17	0.16	0.09	0.08	0.18
Detectable difference for men	0.04	0.05	0.26	0.04	0.02	0.22


## Appendix

Table A.1: Minimum detectable difference in adequacy rates across the WEAI sub-domains, by gender.*Source:* Authors’ calculations. *Note:* These power calculations were based on the original evaluation design with three arms and eight clusters per arm.

## Footnotes

1 The Gender, Agriculture, and Assets Project, Phase 2 (GAAP2) brings together 13 agricultural development projects with explicit women’s empowerment goals, working together to develop and pilot a project-level measure of women’s empowerment and to use it in impact evaluations to assess which strategies work to empower women. For more information, see gaap.ifpri.info.

2 Here the aggregation is done over individuals such that we obtain a single empowerment score and GPI for a given set of individuals. This implies that these are defined at the sample level.

3 The intrahousehold inequality score is a truncated variable and we acknowledge that linear regression is not ideal. However, our analysis rests on the nearest neighbor matching method which does not rely on linear regression.

4 Since the power calculations were based on the study design of the overall evaluation with three arms and eight clusters per arm these should be viewed as suggestive for this analysis, which investigates the impact of SHG membership and not the design of the larger study. Nonetheless, Table A.1 provides the minimum detectable differences across WEAI sub-domains for women and men for a sample of this size.

5 These approaches rely on two assumptions about the data and the model. The first is that, after controlling for all pre-program observable respondent, household and community characteristics that are correlated with SHG membership and the outcome variable, nonmembers have the same average outcome as SHG members would have had if they had not become members. The second assumption is that for each SHG member and for all observable characteristics, a comparison group of nonmembers with similar observable variables exists.

6 We estimated the NNM models using the ‘teffects’ suite of commands in STATA (STATACORP (2015)).

7 This is measured as the time spent by the adult women within the household fetching water from a distant source in summer or winter and is not constructed using the time use module in the WEAI. This is a proxy indicator for women’s time use, as well as for the household’s social and economic status. For example, proximity to and ability to draw water from a handpump or well is often restricted for households of lower social status. To allay concerns that the indicator used for matching might be highly correlated with the time use domain outcome measure, we check the strength of their association – the correlation between the two is very low at 0.006/0.009 (depending on the measure of time use employed). We also note that our results do not change materially if this indicator is dropped.

8 Since districts are entirely contained within state boundaries, we do not include state dummies in the matching exercise.

9 Because pro-WEAI was still being developed, we did not collect the autonomy over income and the self-efficacy indicator in this survey.

10 These estimates are same as the estimates for the corresponding indicators for pro-WEAI because the indicator construction is identical.

11 We do not report the results on indicators that do not change; these are available upon request.
